# Rolling shutter-resistant confocal endomicroscopy image stitching via dual-path Gaussian U-Net

**DOI:** 10.1117/1.JBO.30.12.126001

**Published:** 2025-12-02

**Authors:** Yuhua Lu, Shangbin Chen, Qian Liu

**Affiliations:** a Huazhong University of Science and Technology, Wuhan National Laboratory for Optoelectronics, Wuhan, China; b Hainan University, School of Biomedical Engineering, Key Laboratory of Biomedical Engineering of Hainan Province, Hainan, China

**Keywords:** confocal endomicroscopy, image stitching, U-Net, rolling shutter effect

## Abstract

**Significance:**

Confocal endomicroscopic image stitching can expand the field of view and improve examination efficiency. However, due to interference from the rolling shutter effect, traditional stitching methods may produce misalignments, leading to structural distortion and artifacts. Suppressing the rolling shutter effect in confocal endomicroscopic images can effectively enhance stitching quality.

**Aim:**

We propose a Dual-Path Gaussian U-Net (DGU-Net)-based framework for confocal endomicroscopic image stitching. The parallel dual-encoder paths of DGU-Net extract Gaussian features and conventional features at different resolutions, respectively, achieving more precise gland segmentation masks. Based on these masks, we filter stable frames and optimize feature matching to effectively suppress rolling shutter interference and improve stitching quality.

**Approach:**

We annotated a segmentation dataset comprising 80 rat confocal laser endomicroscopy (CLE) images to train the segmentation network and validated the frame selection method’s effectiveness in suppressing the rolling shutter effect on consecutively acquired rat CLE video sequences. The stitching results generated from the filtered stable image sequences were compared with conventional methods.

**Results:**

Experimental results demonstrate that DGU-Net achieves superior performance with a Dice score of 85.17 on CLE datasets, significantly outperforming existing segmentation networks. Compared with Auto-Stitching, our method improves regional consistency across the panoramic image by eliminating artifacts caused by mismatches while delivering enhanced stitching accuracy and image quality.

**Conclusions:**

The proposed method effectively accomplishes confocal image stitching tasks, significantly enhancing endomicroscopic examination efficiency and contributing to improved diagnostic outcomes.

## 1 Introduction

Colorectal cancer, gastric cancer, and esophageal cancer are all major threats to human health. They account for 16.1% of all new cancer cases and 20.7% cancer-related deaths.^1^ Early diagnosis is crucial, as 5-year survival rates for early-stage cancers are over 90% compared with less than 20% for advanced carcinomas. Endomicroscopic biopsies remain the gold standard in confirming neoplastic tumors and staging progression. However, their invasive nature and delayed feedback contribute to a non-negligible rate of missed diagnoses and false negatives. Confocal laser endomicroscopy (CLE), which allows real-time visualization at the micron level of mucosal cellular structures and subcellular structures, overcomes these limitations. It facilitates precise lesion location and “optical biopsy” capabilities.^2^^–^^5^

**Fig. 1:**
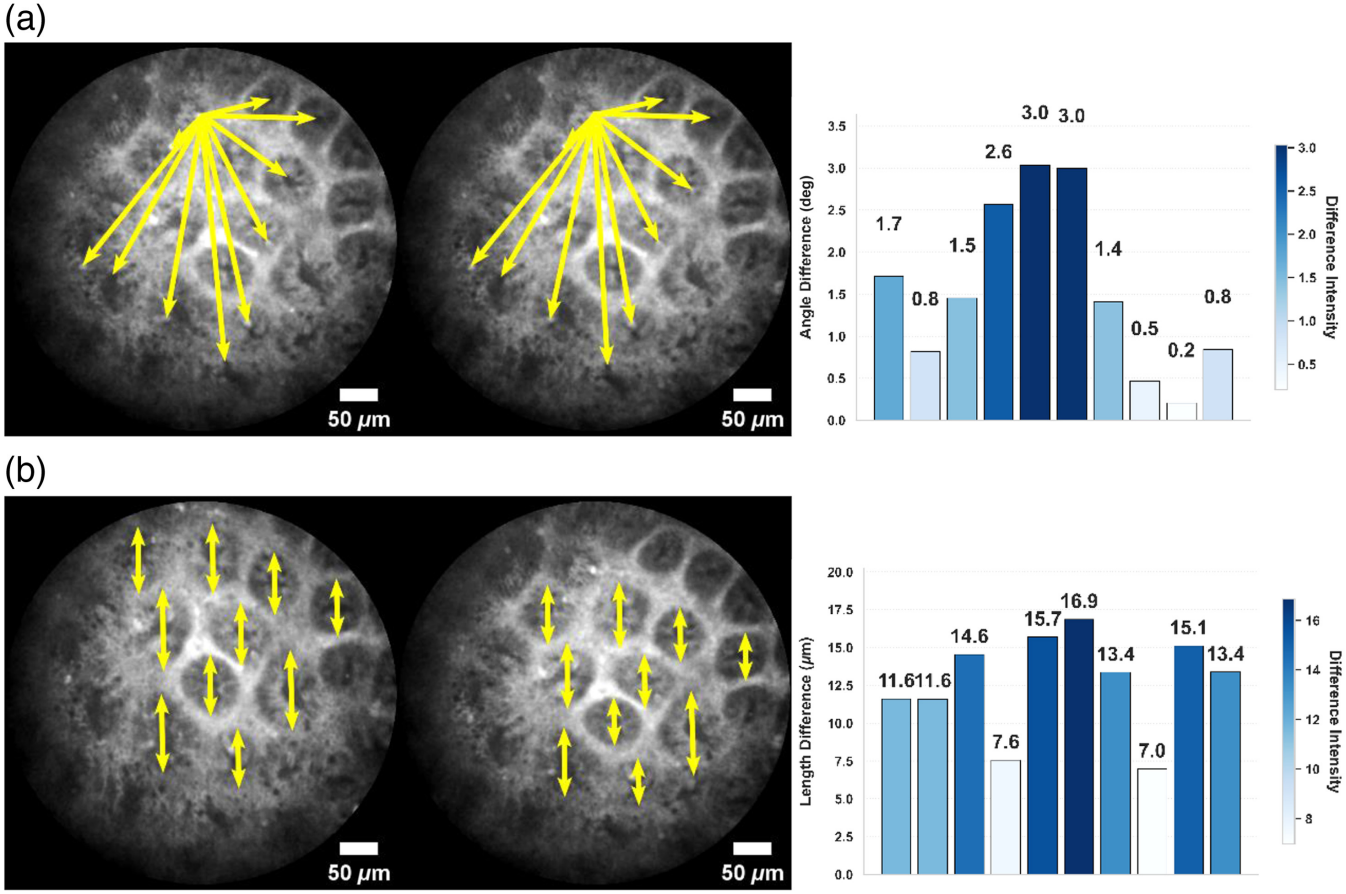
Rolling shutter effects inducing inconsistent distortions between adjacent frames: (a) nonuniform angular displacement in frames 36 (left) and 37 (middle). Single-headed yellow arrows show angular changes of glandular structures relative to a common reference point. The histogram (right) displays angular differences for glandular structures between the two frames; (b) differential glandular deformation in frames 43 (left) and 44 (middle). Double-headed arrows indicate length variations of glandular structures. The histogram (right) displays length differences for glandular structures between the two frames.

Confocal endomicroscopic image stitching can significantly expand the examination field of view, enabling clinicians to more comprehensively assess lesion areas, thereby improving diagnostic efficiency and accuracy.^6^ However, due to the progressive line-scanning mechanism of CLE,^2^ probe movement during imaging introduces rolling shutter effects. As shown in Fig. 1, in adjacent frames, nonsynchronous imaging of different regions causes nonuniform deformation and stretching of glands at different positions. These distortions lead to parallax artifacts and feature matching errors during stitching, significantly compromising final image quality.^7^^,^^8^ Therefore, developing effective rolling shutter suppression algorithms is crucial for enhancing the clinical utility of confocal endomicroscopic image stitching.

Traditional image stitching methods typically rely on feature point matching and geometric transformations. Brown and Lowe^9^^,^^10^ pioneered Scale-Invariant Feature Transform (SIFT)–based global homography alignment for panoramic image generation in computational image stitching, laying the foundation for commercial applications. Subsequent research focused on hardware advancements and feature descriptor optimizations, including Principal Component Analysis-SIFT (PCA-SIFT),^11^ Speeded-Up Robust Features (SURF),^12^ and Features from Accelerated Segment Test (FAST).^13^ However, the global unresponsive matrix alignment method based on feature point matching requires that the optical centers of the images involved in the stitching coincide; otherwise, parallax will occur to fail to align the pictures. To solve this problem, Gao et al.^14^ introduced dual-plane homography by separating scenes into foreground and background layers. Further, Lin et al.^15^ rely on a plurality of affine transformations to align the pictures, giving the image stitching some ability to handle parallax. Zaragoza et al.^16^ proposed dense grid-based homography adaptation to mitigate parallax and distortion artifacts. Zhang et al.^17^ improved the stitching performance of large parallax scenes based on a video de-jittering method and seam line domination. Gao et al.^18^ estimated the optimal geometric transformation using the seam-cut method. Li et al.^19^ obtained the optimal seam line using automatic quaternions.

The advent of deep learning catalyzed paradigm shifts in image stitching, with convolutional neural networks (CNNs) being progressively adapted for this domain. Yi et al.^20^^–^^22^ pioneered LIFT-Net, a tripartite neural framework comprising a dedicated subnetwork for feature point detection, orientation estimation, and descriptor generation. Subsequently, Han et al.^23^ developed MatchNet, which employs a dual-tower architecture for feature extraction, followed by similarity computation through three cascaded fully connected layers. DeTone et al.^24^ introduced HomographyNet, leveraging VGG-inspired architectures to regress the eight parameters of homography matrices directly. Simultaneously, Nie et al.^25^ proposed a rectangling network to eliminate irregular boundaries caused by homography transformations. Jiang et al. integrated adversarial attacks into the stitching pipeline to enhance robustness, whereas Cai et al.^26^ incorporated geometric constraints for natural image stitching applications. However, most of these methods rely on the stability of feature point detection without fully considering the impact of rolling shutter effects on feature detection.

In endomicroscopic image stitching, Vercauteren et al.^6^ pioneered diffeomorphic modeling for confocal image reconstruction, which was subsequently integrated into the commercial CLE system developed by MKT. Building upon this imaging platform, Gong et al.^27^ proposed a context-aware registration algorithm (CWCR) that combines feature-based matching with template-based rigid alignment to achieve global frame registration. Kose et al.^28^ implemented video stitching through SIFT feature extraction and graph-cut optimization. Rosa et al.^29^ developed a servo motor-controlled probe positioning system, where image registration was guided by precise motor position feedback. Bedard et al.^30^ introduced a cost-effective discrete Fourier transform-based approach for frame alignment in a custom high-resolution endomicroscopic system. In gastroscopic applications, Zhou et al.^31^ achieved real-time stitching through stable frame selection combined with SURF feature matching. Zenteno et al.^32^ enhanced stitching robustness by employing optical flow-based image selection to minimize texture discontinuities. These methodologies demonstrate systematic optimization of stitching algorithms tailored to specific endomicroscopic imaging characteristics.

In this paper, we propose a novel stitching technique for the gastrointestinal CLE imaging system developed by our research group.^2^ We developed a Dual-Path Gaussian U-Net (DGU-Net) that extracts Gaussian and non-Gaussian features at different resolutions through its dual-path mechanism, enabling better perception of textural features in gastrointestinal CLE images for more accurate segmentation mask generation. The segmentation masks are then used to filter stable frames from the image sequence and extract foreground features, thereby suppressing rolling shutter effects. Finally, we utilize the foreground SIFT features of the selected stable frame sequence to generate stitched images. The main contributions to this work can be summarized as follows:

1. Constructed a meticulously annotated segmentation dataset comprising 80 gastrointestinal CLE images and developed a dedicated gland segmentation network for digestive endomicroscopy
2. Developed a segmentation mask-based frame selection method that identifies stable frames in gastrointestinal CLE image sequences, effectively suppressing rolling shutter effects
3. Successfully implemented gastrointestinal confocal stitching using our proposed method. Comparative experiments were conducted with conventional methods to demonstrate the superiority of our approach.

The remainder of this paper is organized as follows: Section 2 details the proposed methodology. Section 3 presents experimental validation and results. Section 4 discusses limitations and future directions. Section 5 concludes by summarizing key contributions and highlighting potential clinical applications of the proposed framework.

## 2 Materials and Methods

The CLE imaging system employs a galvanometer-based XY scanning mechanism with counter-rotating mirrors for row-wise acquisition.^2^ Each frame is sequentially captured by scanning individual rows while progressively advancing columns along the Y-axis. Probe movement during this scanning process generates nonuniform spatial displacements—particularly evident in glandular structures—manifesting as rolling shutter effects.

**Fig. 2:**
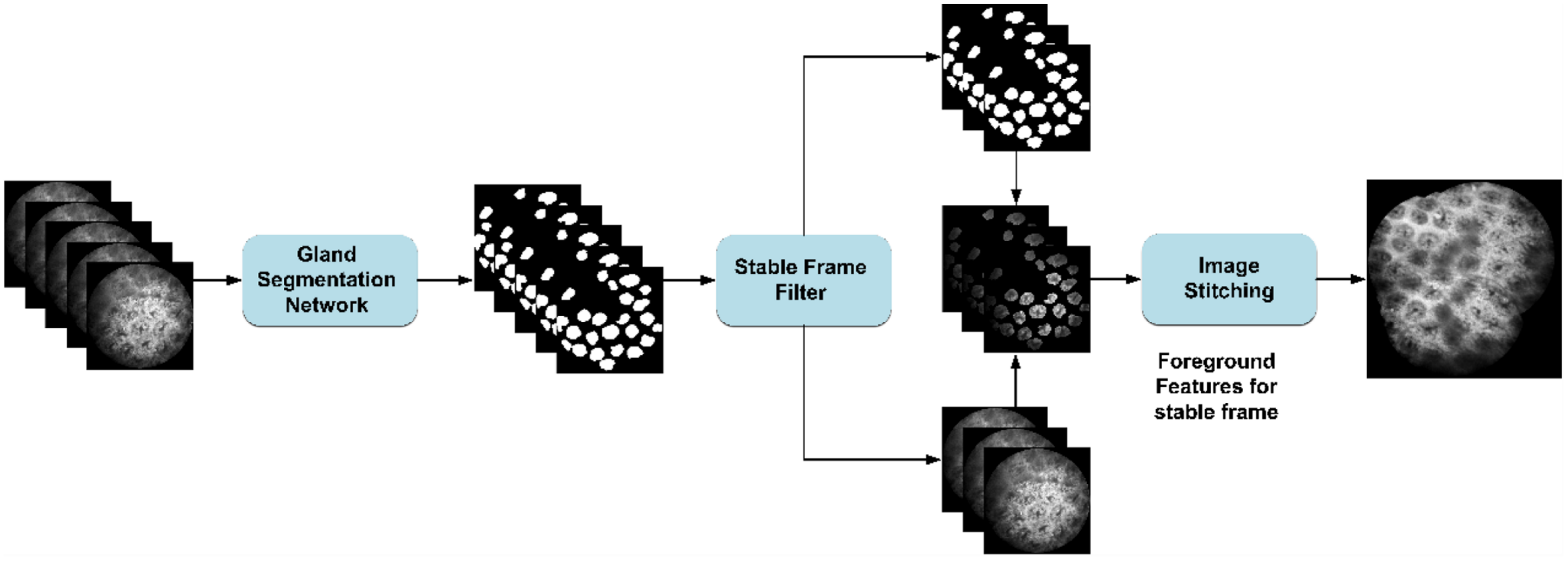
Confocal endomicroscopic image stitching pipeline, consisting of three key stages: (1) a gland segmentation network trained to generate binary masks isolating diagnostically relevant regions, (2) stable frame selection by minimizing inter-frame projection differences using segmentation masks, and (3) SIFT descriptors extracted exclusively from stabilized foreground regions enable robust feature matching and seamless image alignment.

We developed a novel image stitching framework based on a gastrointestinal gland segmentation network, as shown in Fig. 2, to suppress rolling shutter effects and improve stitching accuracy.

**First**, we trained a gland segmentation network on CLE images to generate binary masks isolating glandular foregrounds from background interference. **Subsequently**, these masks guided frame stabilization through temporal consistency analysis, where sequential frames were aligned using mask-based projective differences to compensate for rolling shutter effects. **Finally**, SIFT descriptors were exclusively extracted from stabilized glandular regions to enable robust feature matching, followed by multi-band blending to generate seamless panoramic images.

### 2.1 Segmentation Network

**Fig. 3:**
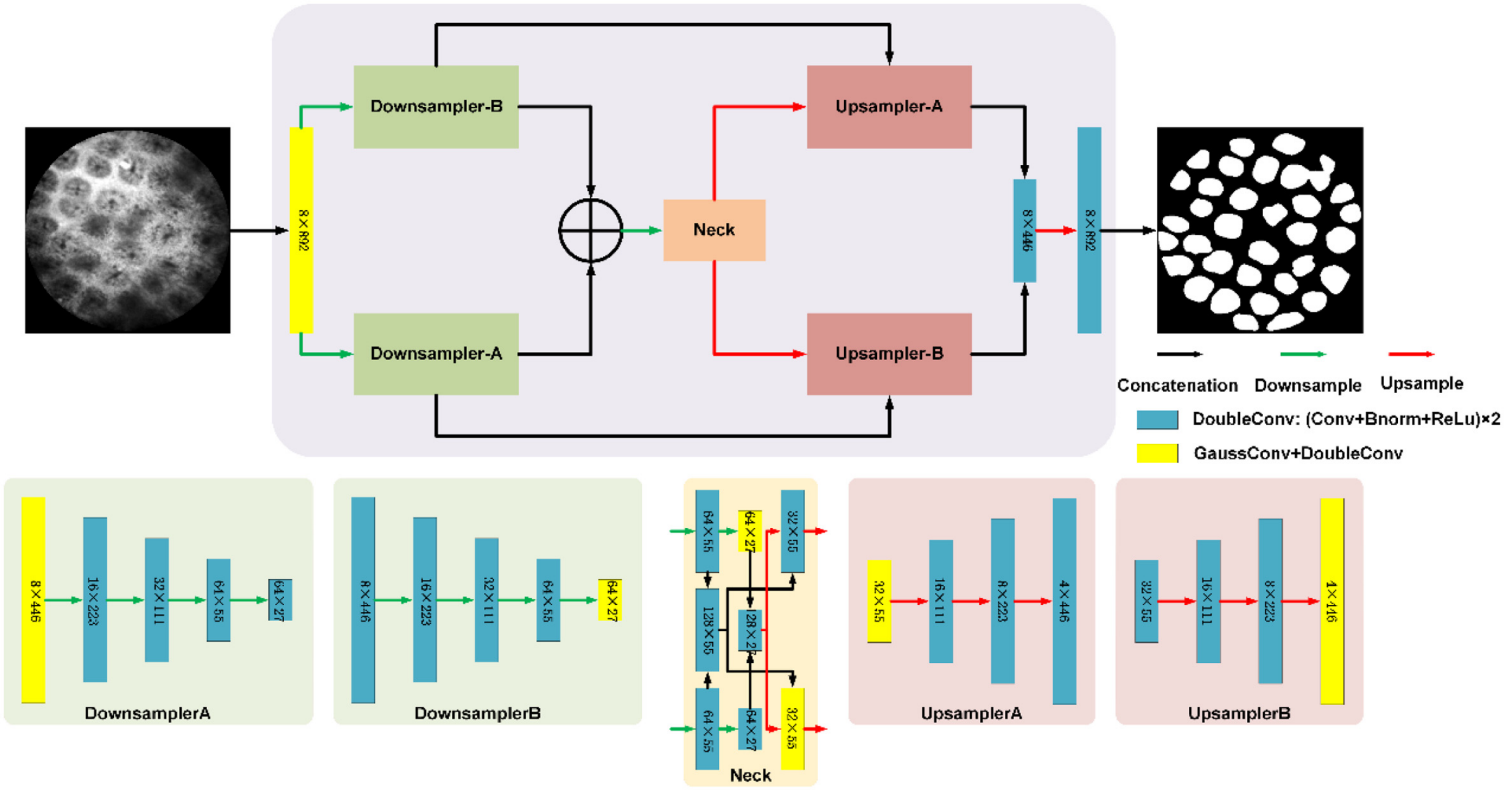
Architecture of the Dual-Path Gaussian U-Net with 892×892  pixel input and output dimensions. Blue blocks represent DoubleConv layers with 3×3 convolutional kernels, whereas yellow blocks denote GaussDoubleConv layers incorporating a 9×9 Gaussian convolutional kernel before the DoubleConv layer. Black arrows indicate data flow, green arrows represent downsampling layers, red arrows denote upsampling layers, and blue arrows signify concatenation operations.

As shown in Fig. 3, we designed a DGU-Net that integrates Gaussian smoothing and dual-path mechanisms to enhance feature extraction and segmentation performance.^33^^–^^36^ The dual-path mechanism processes features through two parallel streams, which are later fused to capture both local texture details and global contextual information.

#### 2.1.1 Gaussian convolution

The proposed architecture relies on Gaussian convolutional layers. These layers apply a fixed Gaussian kernel to the input feature map, smoothing the data while maintaining structural information. The 2D Gaussian kernel is defined as a size:

$$G(x,y)=\frac{1}{2\pi \sigma^{2}}\;\exp(-\frac{(x-\mu {)}^{2}+(y-\mu {)}^{2}}{2\sigma^{2}}),$$ (1)

where (x,y) represents the spatial coordinates, μ is the mean (centered at the kernel’s midpoint), and σ is the standard deviation. The kernel is normalized to ensure that the sum of its weights equals one.

#### 2.1.2 Encoder

After initial Gaussian convolution preprocessing, feature maps enter the five-stage progressive encoder structure. The first path applies Gaussian convolution before the initial downsampling, whereas the second path maintains original feature processing until the fifth downsampling stage, extracting both Gaussian and non-Gaussian features at different resolutions. Feature concatenation occurs at the fourth and fifth downsampling levels of both paths, enabling fusion of local details with global features. This design preserves U-Net’s multi-scale extraction advantages while enhancing feature diversity through differentiated dual-path processing.

#### 2.1.3 Decoder

The decoder mirrors the encoder’s structural design. The first upsampling path employs Gaussian convolution in its initial upsampling, whereas the second path applies Gaussian processing at the fourth upsampling stage—balancing detail preservation with feature smoothing to ensure edge continuity in final outputs. Following feature concatenation of both paths, a final upsampling restores feature map dimensions before two convolutional layers generate the segmentation mask.

### 2.2 Stable Frame Selection

Clinical CLE examinations follow a “slow translation-brief pause” model where operators intermittently move and stabilize the probe. This generates alternating sequences of roll shutter-distorted frames and stable frames. Stabilizing frame selection is critical for mitigating rolling shutter-induced distortions in image stitching.

The gland segmentation network was employed to generate binary masks for each frame. Structural variations were quantified through row-wise and column-wise projection analysis of these masks: The row projection R(y) and column projection C(x) are defined as

$$R(y)=\sum_{x=1}^{W}M(x,y),$$ (2)

$$C(x)=\overset{H}{\underset{y=1}{\sum }}M(x,y),$$ (3)

W and H denote image width and height, respectively, and represent the binary segmentation mask.

The projection difference metric D(i,i+1) between consecutive frames was computed as

$$D(i,i+1)=\overset{H}{\underset{y=1}{\sum }}(R_{i}(y)-R_{i+1}(y){)}^{2}+\overset{W}{\underset{x=1}{\sum }}(C_{i}(x)-C_{i+1}(x){)}^{2},$$ (4)

where $R_{i}(y)$ and $R_{i+1}(y)$ denote row projections of frames i and i+1, respectively, whereas $C_{i}(x)$ and $C_{i+1}(x)$ represent their column projections. Frames are classified as stable if D(i,i+1), all are below an empirically determined threshold and discarded as motion-corrupted frames. This value corresponds to the optimal separation point that maximizes discrimination between lower-value clusters (indicating stable frames) and higher-value clusters (indicating motion-corrupted frames) in the characteristic bimodal distribution. In this work, this threshold was set to 0.8.

### 2.3 Image Stitching

The stabilized frames and their foreground features were processed through the following pipeline.

#### 2.3.1 Feature extraction

Mask-constrained SIFT features were extracted by filtering keypoints using gland segmentation masks. Specifically, a binary mask is used to constrain the SIFT feature extraction process. Only keypoints located within the foreground region (where the mask value is 1) are retained, whereas keypoints in the background region are discarded. This ensures that the extracted features are relevant to the foreground objects and significantly reduces the impact of background clutter on the stitching process. SIFT descriptors (128-dimensional feature vectors encoding local gradient histograms) are computed at all detected keypoints. Subsequently, the set of filtered keypoints $K_{\text{filtered}}$ is defined as

$$K_{\text{filtered}}=\{(x,y)|M(x,y)=1\},$$ (5)

where (x,y) are the coordinates of the keypoints detected by SIFT, M(x,y) are the keypoints that are within the segmentation masks. This filtering step ensures that only keypoints within the foreground region are considered for further processing.

#### 2.3.2 Feature matching

After extracting all features from the images, we use the FLANN-based matching algorithm and Lowe’s Ratio Test to find correspondences. The ratio test filters ambiguous matches out by retaining only matches where the distance between the best and second best matches is below a threshold (ratio = 0.75). This step produces reliable feature correspondences for image pairs that are used to estimate geometric transformations between images.

#### 2.3.3 Homography estimation

We estimate the homography matrix for each image pair using the matched feature points. The homography matrix represents the geometric transformation (e.g., rotation, translation, scaling) that aligns one image with another. Random Sample Consensus (RANSAC) is used to handle feature matches. RANSAC selects a subset, computes the homography, and evaluates its consistency. The homography that has the most inliers will be chosen as the final transform. This robust estimation ensures that alignment is accurate even when features are noisy or mismatched.

#### 2.3.4 Gain compensation

We apply a gain-compensation algorithm to address brightness inconsistencies between images. The algorithm minimizes differences in intensity between overlapping regions of image pairs. It solves a system of linear equations to calculate a gain factor per image. The gain factors are then applied to the images to ensure seamless blending of the final panorama. This step is crucial for producing visually consistent results.

#### 2.3.5 Multi-band blending

Finally, the aligned pictures are blended together into a single panoramic image using a multi-band blend. This technique divides each image using Gaussian Pyramids into multiple frequency bands and blends them separately. The low-frequency band is blended over a large spatial range to smooth out color and brightness differences, whereas the high-frequency band is blended over a small range to preserve fine detail. The blended bands are recombined to create the final panorama. This ensures that the final result will be visually seamless and full of detail.

### 2.4 Dataset

A custom fiber-optic CLE probe was used to image excised rat stomach tissues with DSS-induced ulcerative gastritis. System specifications included a 488-nm excitation laser, an intravenous fluorescein sodium contrast agent, 1.4-μm lateral resolution, 512.5×512.5  μm FOV, five fps acquisition rate, and 892×892  px image size. The dataset comprises 400 frames acquired from two rats (200 frames per rat). From these, 80 images were randomly selected for segmentation network annotation, with multiple glandular structures annotated in each image. Ground truth segmentation masks were meticulously annotated by two experienced researchers under the supervision of a senior endoscopist. All imaging experiments were approved by the animal experiment guidelines of the Animal Experimentation Ethics Committee of Huazhong University of Science and Technology (HUST, Wuhan, China).

## 3 Result

### 3.1 Segmentation Performance

In this section, experimental results are presented of our **DualPath Gaussian UNet (Ours)**, which we have proposed for gland segmentation on confocal endomicroscopy of the digestive system. To evaluate the effectiveness of our approach, we conducted extensive experiments including ablation studies, comparisons with other segmentation methods, and comparisons to state-of-the-art segmentation methods. Results are reported based on five widely used evaluation metrics: accuracy (AC), Dice coefficient (Dice), Jaccard Index (JA), sensitivity (SE), and specificity (SP).

#### 3.1.1 Ablation study

To compare the various variants of our proposed network and demonstrate their effectiveness, we conducted a study on ablation.

U-Net: U-Net is the baseline architecture.

GU-Net: A U-Net variant incorporating Gaussian convolution (GaussianConv2d) instead of standard convolutions.

DU-Net: A U-Net variant introducing a dual-path structure but without Gaussian convolution.

Ours (DualPath Gaussian UNet): We propose a network that combines Gaussian Convolution with a dual-path architecture.

**Fig. 4:**
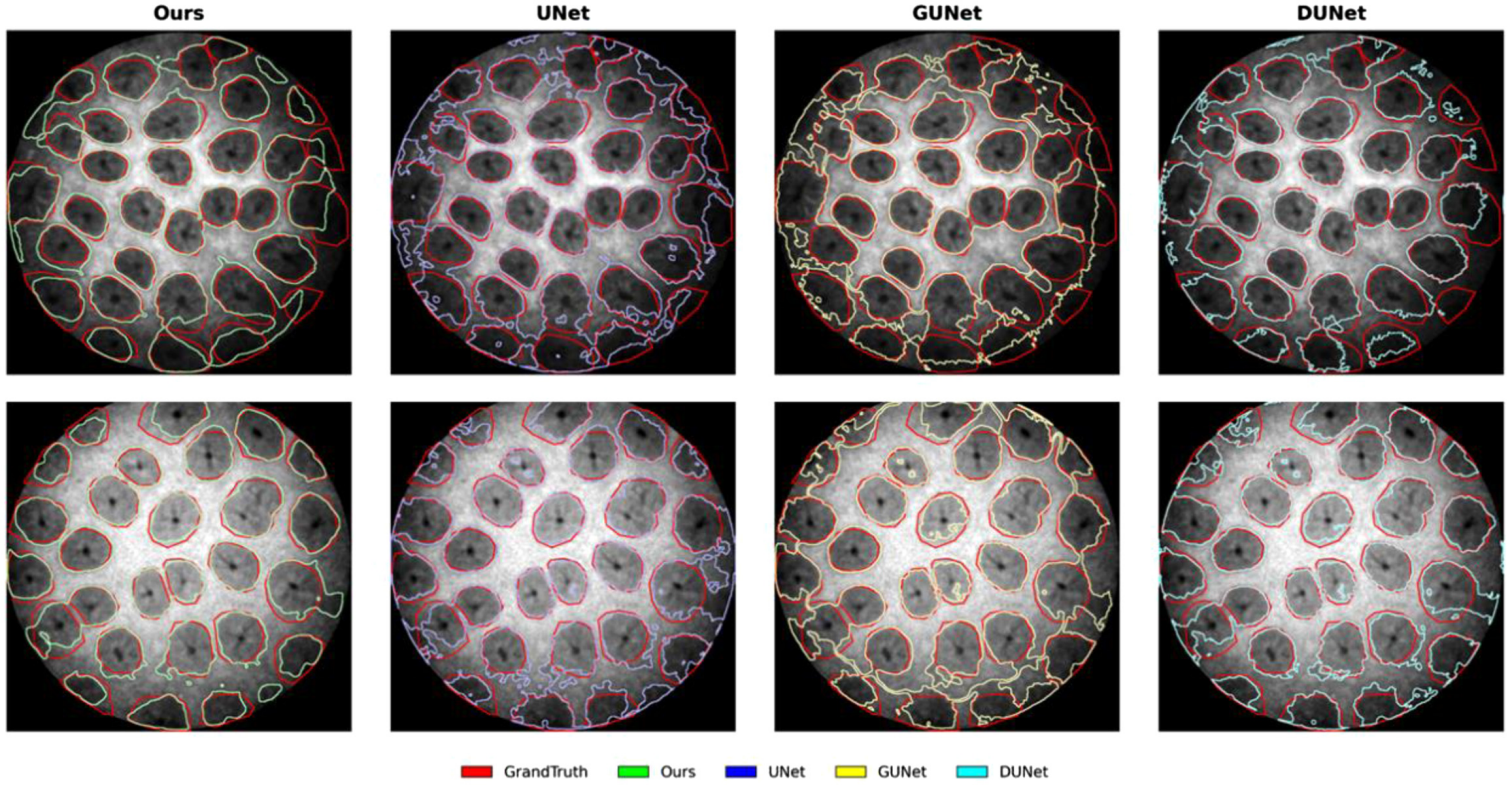
Ablation study results, presenting contour comparisons between the proposed method, variant networks, and ground truth.

The results of the ablation study are summarized in Table 1. Our proposed DualPath Gaussian U-Net achieves superior performance on key segmentation metrics, with an AC of 88.10, a Dice of 85.17, JA of 74.75, and SE of 87.03, significantly outperforming other variants. Although the specificity (SP, 88.83) is marginally lower than DU-Net (89.79), the overall performance demonstrates the synergistic effect of Gaussian convolution and dual-path structure in enhancing the network’s capability to segment fine glandular structures. Notably, GU-Net underperformed the baseline U-Net due to excessive smoothing, whereas DU-Net’s higher Dice and JA scores confirm the effectiveness of the dual-path architecture. As shown in Fig. 4, the segmentation results for the proposed method and the variant networks are presented.

**Table 1:** Ablation study of different network components.

Methods	AC	Dice	JA	SE	SP
U-Net^37^	86.25	83.03	71.66	85.91	86.48
GU-Net	85.41	82.16	70.32	85.34	85.47
DU-Net	87.45	84.89	73.04	84.01	**89.79**
Ours	**88.10**	**85.17**	**74.75**	**87.03**	88.83

The best results are highlighted in bold.

#### 3.1.2 Comparison with state-of-the-art methods

We compared our proposed DualPath Gaussian U-Net with several state-of-the-art segmentation methods, including U-Net,^37^ U-Net++,^38^ U-Net3+,^39^ Attention U-Net (Att U-Net),^40^ Adaptive Attention U-Net (AAU-Net),^41^ and SegNet.^42^ The results are presented in Table 2.

**Table 2:** Segmentation results of different methods.

Methods	AC	Dice	JA	SE	SP
U-Net^37^	86.25	83.03	71.66	85.91	86.48
U-Net++^38^	87.09	84.23	73.26	87.36	86.90
U-Net3+^39^	87.10	84.11	73.15	86.82	87.29
Att U-Net^40^	77.54	76.51	62.72	**93.33**	66.80
AAU-Net^41^	84.99	81.64	69.74	85.47	84.66
SegNet^42^	83.43	80.05	67.42	84.47	82.57
Ours	**88.10**	**85.17**	**74.75**	87.03	**88.83**

The best results are highlighted in bold.

**Fig. 5:**
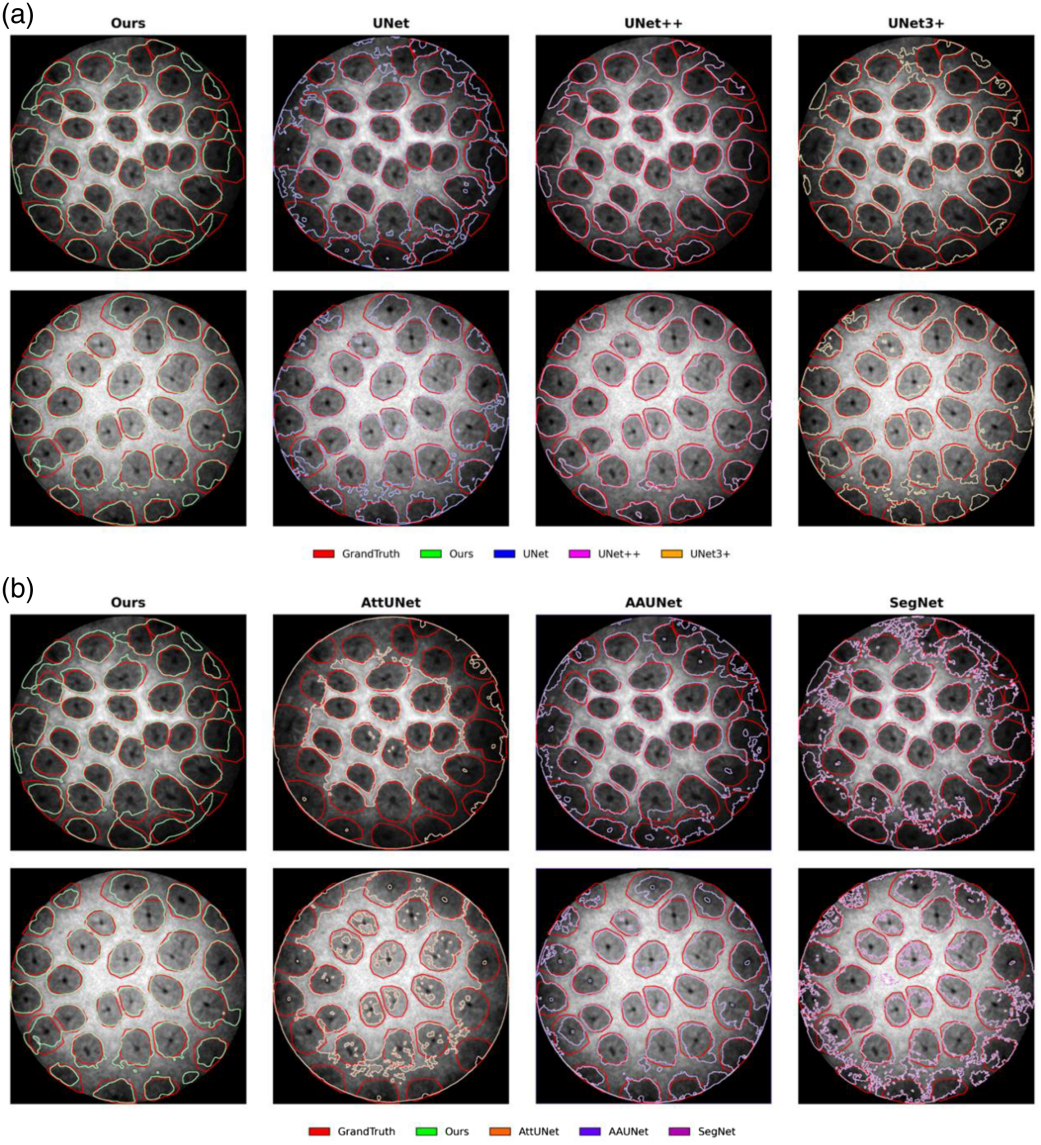
Comparison study results. For clarity, competing methods are divided into two groups, each showing contour comparisons between the proposed method, three competing methods, and ground truth: (a) UNet, UNet++, and UNet3+; (b) AttUNet, AUUNet, and SegNet.

Our method outperforms the second-best performer across most evaluation metrics. We achieved improvements of +1.0 AC,+0.94 Dice, and +1.49 JA over the second-best performer. As shown in Fig. 5, the proposed method achieves a cleaner segmentation boundary than other approaches. These results demonstrate the superiority of our approach when handling the complex structures and varying intensity present in confocal endomicroscopy images.

#### 3.1.3 External test

To validate the generalization capability of our approach, we evaluated the segmentation network on an external breast ultrasound dataset comprising 42 images, each containing an annotated breast tumor region and uniformly resized to 128×128  pixels as network input.^43^ Compared with gastrointestinal confocal endomicroscopy images, breast ultrasound images differ in texture patterns and lesion morphology, yet share similar segmentation challenges in distinguishing salient structures from noisy backgrounds, making them an appropriate benchmark for evaluating our model’s universal feature extraction capability. As shown in Table 3, the proposed method achieved the highest accuracy (AC) and Jaccard Index (JA) among all evaluated networks. Although not optimal in all metrics, its performance across different medical imaging modalities confirms the generalization of the dual-path Gaussian architecture for segmentation tasks.

**Table 3:** Segmentation performance comparison on the external breast ultrasound dataset.

Methods	AC	Dice	JA	SE	SP
U-Net^37^	97.08	**92.81**	83.31	87.55	98.47
U-Net++^38^	97.73	89.28	83.39	93.74	98.28
U-Net3+^39^	97.68	89.35	82.44	89.68	98.79
Att U-Net^40^	97.50	87.24	81.00	87.82	**98.83**
AAU-Net^41^	97.58	88.14	81.64	88.75	98.80
SegNet^42^	96.70	87.08	77.04	87.01	98.11
Ours	**98.13**	91.41	**85.92**	**94.07**	98.69

The best results are highlighted in bold.

#### 3.1.4 Experiment details

The loss function for network training is binary-cross-entropy. Adam optimizer is used to train the network. The initial learning rate is set at 0.00005. The dataset is randomly split into training (80%) and validation (20%) sets using a fixed random seed to ensure reproducibility. Multiple cross-validation shows that the best segmentation performance is obtained when epoch size and batch size are set to 100 and 2, respectively. The development environment consists of Pytorch with CUDA acceleration 12.1, Python 3.9, and an NVIDIA RTX3090 GPU.

### 3.2 Stabilization Performance

In this section, we present the results from the frame selection method for mitigating rolling shutter effects in confocal image sequences of the digestive system. We employed the video stabilization evaluation framework proposed by M. Grundmann^44^ to assess the improvement in stability of the stabilized frame sequence compared with the original sequence by estimating the variations in the camera path.

#### 3.2.1 Experimental protocol

Because the frame selection method removes shaky frames from the original image sequence, the stabilized image sequence has fewer frames. Before conducting the stability evaluation, it is necessary to interpolate frames in the stabilized sequence to match the frame count of the original sequence. We employ the Farneback optical flow^45^ method, with the interpolated frame $I_{\text{interp}}$ is computed as

$$I_{\text{interp}}(x,y)=(1-\alpha )\cdot I_{1}(x^{\prime },y^{\prime })+\alpha \cdot I_{2}(x^{\prime },y^{\prime }),$$ (6)

where $I_{1}(x^{\prime },y^{\prime })$ and $I_{2}(x^{\prime },y^{\prime })$ denote forward- and backward-warped frames via the optical flow field $F(x,y)=(F_{x},F_{y})$ with α∈[01] controlling temporal weighting.

The forward optical flow is remapped as $\left(x^{\prime },y^{\prime })=(x+\alpha \cdot F_{x}(x,y),y+\alpha \cdot F_{y}(x,y)\right)$, and the reverse optical flow is remapped as $\left(x^{\prime },y^{\prime })=(x-1-\alpha \cdot F_{x}(x,y),y-1-\alpha \cdot F_{y}(x,y)\right)$, The interpolation factor α is determined by, where T is the total interpolated frames between two stable frames, and t is the current interpolation index.

Subsequently, the camera path of the image sequence is estimated, and the affine change matrix T is estimated for each neighboring frame, accumulating the neighboring frame transformation matrix $T_{\mathrm{accum}}=T_{1}\cdot T_{2}\cdot \ldots \cdot T_{i}$, and the extraction of translation information $T_{x}$ and $T_{y}$ can be used to plot camera motion trajectories of the original and stabilized image sequence.

#### 3.2.2 Performance comparison

**Fig. 6:**
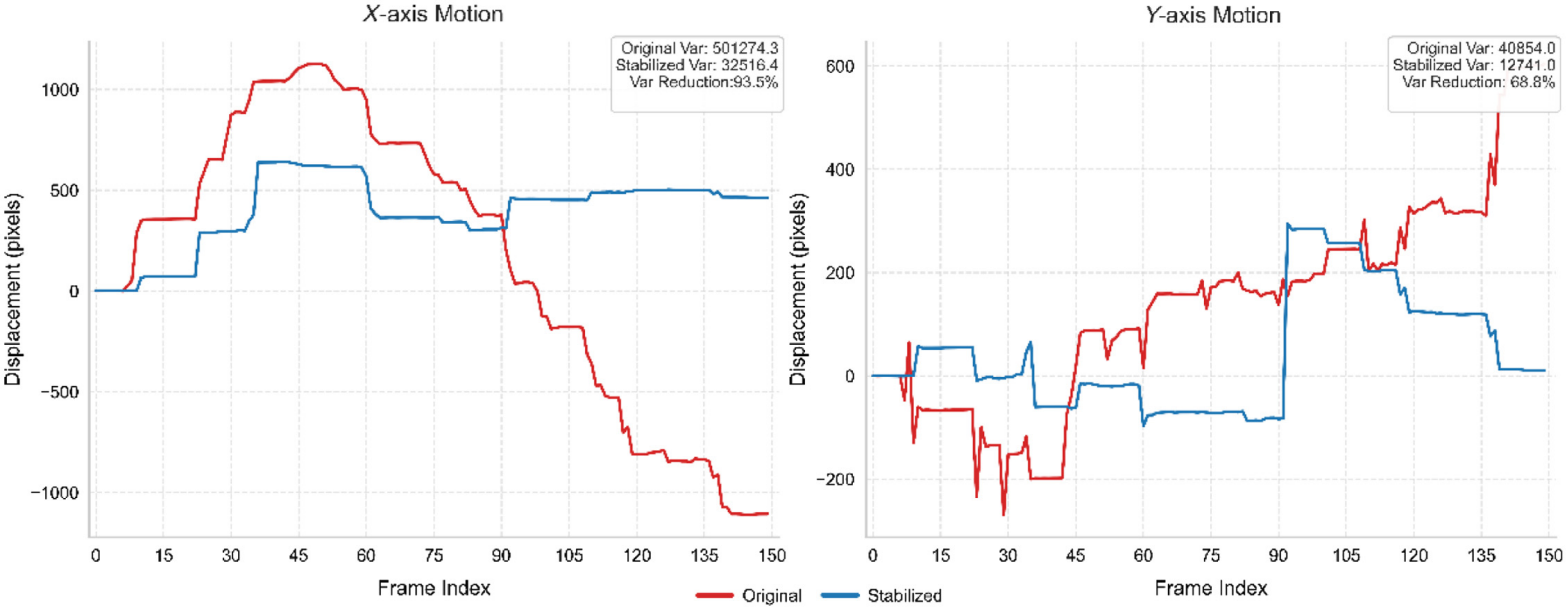
Camera motion paths in the X and Y directions of the original image sequence, and the frame-interpolated stabilized sequence over 150 frames. Our method reduces the jitter of the image sequence.

As shown in Fig. 6, we present the camera paths of the original image sequence and the stabilized image sequence with frame count alignment, showing a reduction in displacement variance along both the Y-axis and X-axis. After stable frame selection and subsequent frame interpolation using the optical flow method to align the frame count, the stabilized image sequence demonstrates lower displacement variance in the X and Y directions than the original sequence. This indicates a jitter reduction and effectively suppresses the rolling shutter effects in the stabilized sequence.

### 3.3 Stitching Performance

This section compares the stitching results between our method and the traditional AutoStitch technique and shows how our method preserves important diagnostic information during confocal endomicroscope inspections of the digestive system.

**Fig. 7:**
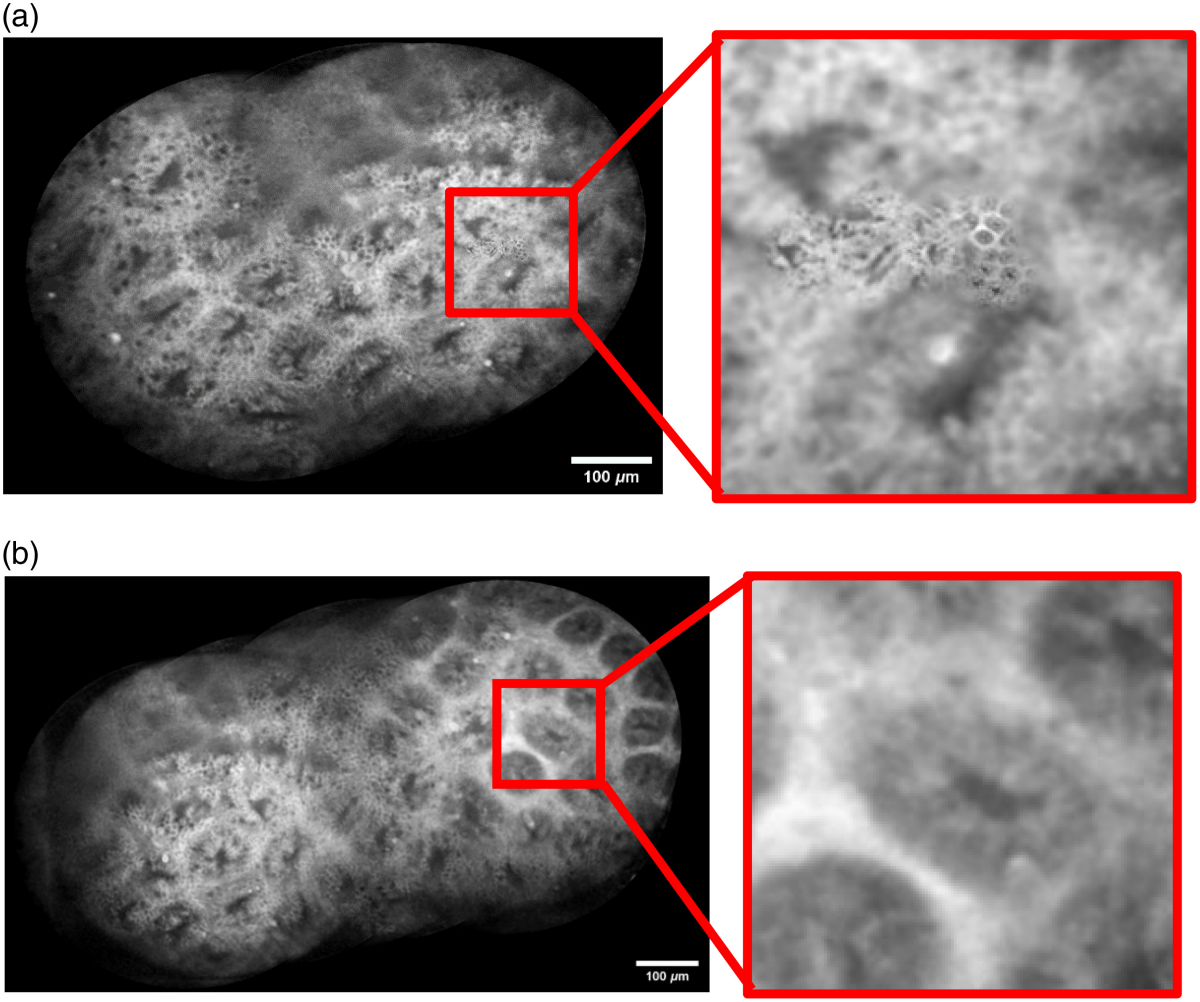
A 50-frame sequence of confocal endomicroscopic images of the gastrointestinal tract: (a) auto-stitching result, showing misalignments; (b) result of our proposed method.

As shown in Fig. 7, for 50 consecutive frames of confocal images of a rat stomach, the AutoStitch method, under the interference of the rolling shutter effects, resulted in incorrect scaling and transformations during image registration, causing some images to be stitched into erroneous positions. By contrast, our method utilizes gland segmentation masks to filter stable frames and foreground feature points, effectively suppressing the rolling shutter effects and background noise. As shown in Fig. 7(b), the resulting stitched images maintain anatomical continuity and eliminate mismatches.

A comparison was made between the proposed method and the conventional Auto Stitch approach. Figure 7 demonstrates stitching outcomes generated from sequential CLE frames of gastrointestinal examinations.

**Fig. 8:**
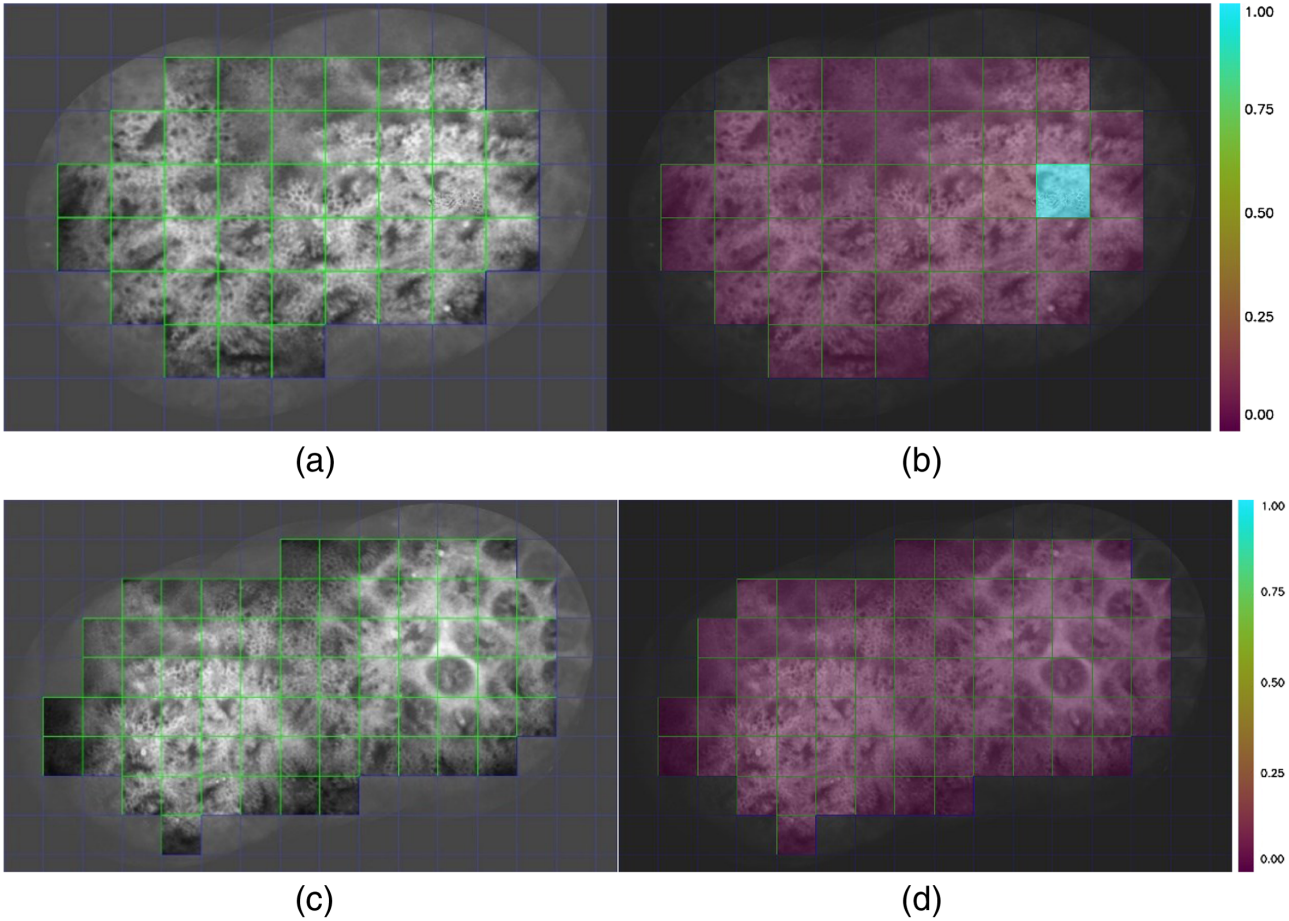
Block-based sharpness consistency evaluation (block size: 128×128  pixels): (a) grid partitioning of AutoStitch result—the stitched image divided into equal-sized blocks, with border regions containing black margins treated as invalid; (b) sharpness heatmap for AutoStitch—individual block sharpness quantified using Laplacian variance, normalized and visualized through a thermal color map (purple indicating lower values, blue indicating higher values); (c) grid partitioning of proposed method’s result under identical partitioning scheme; and (d) sharpness heatmap for proposed method’s result under identical partitioning scheme.

For a sequence of confocal endomicroscopic images of the digestive tract continuously captured by the same device, the clarity of each frame should be consistent, and the resulting stitched image should exhibit more uniform sharpness across all regions. As shown in Fig. 8, we divided the stitched images into several equally sized small patches. We tested the variance of sharpness across these patches at different patch sizes for both our method and the auto-stitching method, as presented in Table 4. A lower variance indicates better consistency in sharpness and higher quality of the stitched image. According to Table 4, under different metrics and patch segmentation sizes, the stitched images generated by our method consistently demonstrate superior uniformity.

**Table 4:** Comparison of variance in image sharpness evaluation metrics across different tile sizes (64×64, 128×128, 256×256  pixels) for stitched images generated by our method and AutoStitch (AS). Lower variance values indicate superior sharpness uniformity.

	Laplacian^46^		Sobel^47^		Brenner^48^	
	Ours	AS	Ours	AS	Ours	AS
64	**133.789**	3120.209	**664,856**	2675004	**745.4142**	908.0533
128	**33.04581**	1878.634	**529,234**	2164628	**409.6784**	821.5077
256	**40.9421**	1035.356	**316,227**	1775584	**476.4396**	1029.241

Methods achieving better consistency (lower variance) are highlighted in bold.

To further evaluate stitching quality on the 100-frame sequence, we employed two complementary metrics: Structural Similarity Index Measure (SSIM) and root mean square error (RMSE), as presented in Table 4. Although the SSIM value of the proposed method was marginally lower than that of Auto-Stitch, this is attributed to the frame selection process, which intentionally excludes motion-distorted frames, leading to increased variations in temporal continuity. Conversely, the proposed method achieved a lower RMSE, indicating superior suppression of large misalignments. These results demonstrate enhanced precision in maintaining diagnostic feature alignment while ensuring clinically acceptable structural integrity (Table 5).

**Table 5:** Comparison of Structural Similarity Index Measure (SSIM) and root mean square error (RMSE) for stitching images generated by our method and Auto-Stitching using a 100-frame sequence. Higher SSIM and lower RMSE indicate superior quality.

Method	SSIM^49^	RMSE^49^
Auto-stitching	**0.8741 ± 0.0761**	44.46 ± 31.63
Ours	0.8461 ± 0.1105	**37.47 ± 21.01**

Methods achieving better performance per metric are highlighted in bold.

**Fig. 9:**
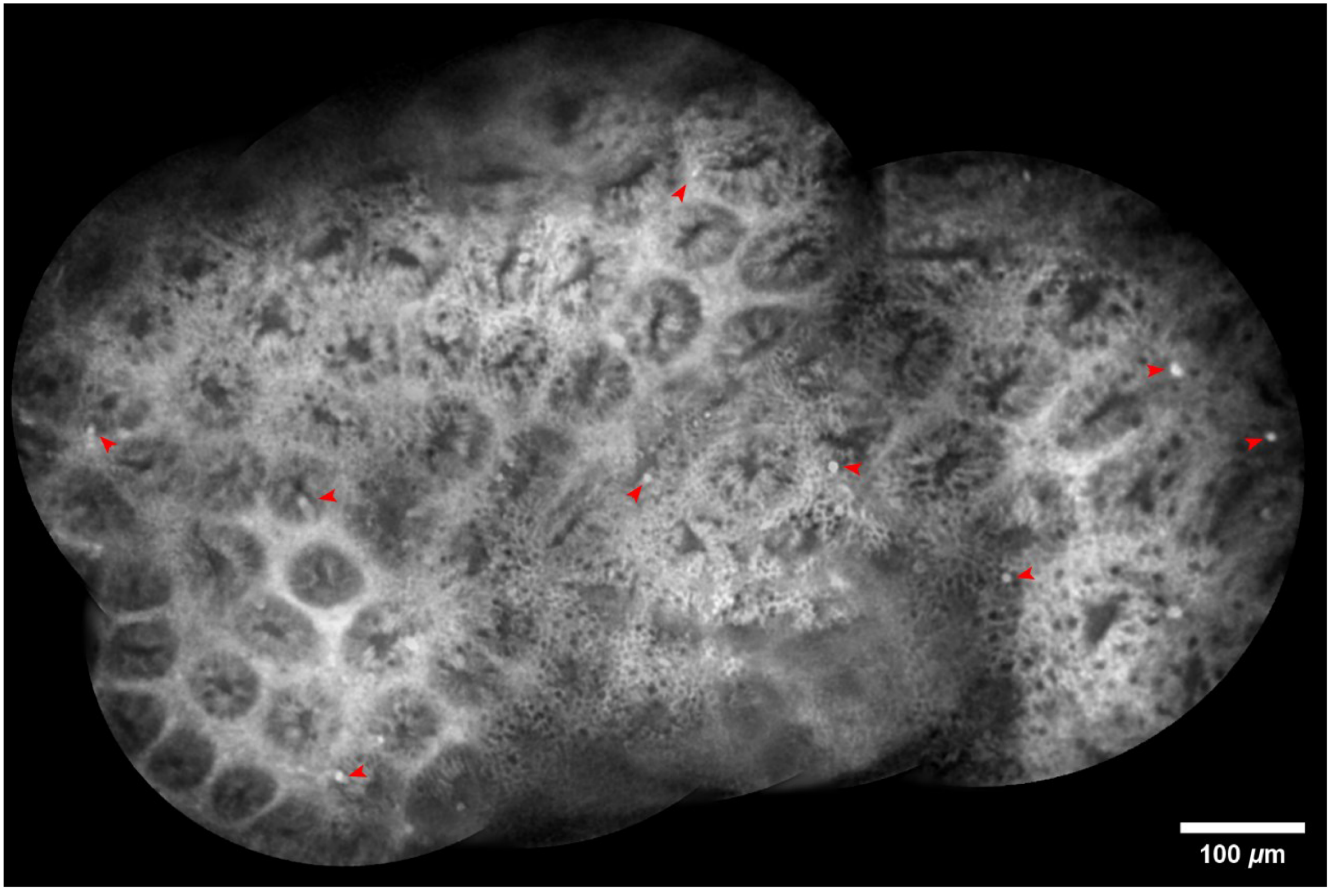
Stitching results of 100 consecutive confocal endomicroscopic images of the gastrointestinal tract. Red arrows indicate observable fluorescein leakage points. (Field of view: 1000×700  μm).

As shown in Fig. 9, our stitching results preserve fluorescein leakage points, a key feature for diagnosing atrophic gastritis in the confocal endomicroscope.^3^ The proposed method, which utilizes gland segmentation masks for stable frame selection and foreground feature extraction, not only improves the accuracy and quality of stitching but also retains critical diagnostic information.

## 4 Discussion

In this paper, a method is presented for stitching confocal pictures of the digestive system based on a U-shaped CNN. During confocal endomicroscopy of the digestive system, disease stage and type diagnosis are based on the characteristics of the superficial cells and glands. The limited field of vision under high magnification in confocal endomicroscopy does not allow for the full lesion to be displayed in a single image. Image stitching can increase the field of view and provide a more accurate and comprehensive representation of the superficial state of the digestive system. This improves the efficiency of clinical examinations and the accuracy of diagnosis. Due to the line scan imaging principle of confocal endomicroscopy, multiple image regions are not captured at once. The rolling shutter effect is caused by the operator moving the probe. This results in mismatched images and affects the image quality.

We use the texture features from confocal images with glandular tissue in the foreground to construct a Dual Path Gaussian UNet for obtaining segmentation masks. The network is able to better perceive texture features in images through Gaussian Convolution and a dual-path structure. As presented in Table 2, we achieve superior segmentation results compared to U-Net networks for confocal images of the digestive system.

As shown in Table 3, the proposed model maintains robust segmentation performance on the external dataset. Although the training dataset comprises only 80 annotated images, each contains multiple glandular structures, resulting in almost 1000 annotated gland instances. This rich morphological diversity enables the model to achieve effective generalization capability despite the limited training volume.

In addition, based on a clinical practice of “slow translation, brief pause,” we use a foreground segmentation to filter out interferences from free superficial cells and a frame selection to remove shaky images while retaining stable frames. The image sequence stabilized from stable frames shows reduced jitter and effectively suppresses the rolling shutter effects.

As shown in Fig. 7, the panoramic image generated through SIFT feature registration of foreground elements in stable frames eliminates mismatches inherent to conventional methods. This approach provides enhanced visualization of glandular morphology and pathological indicators (e.g., fluorescein leakage points) under confocal endomicroscopy, enabling comprehensive assessment of the digestive tract’s superficial state. Clinicians can thereby accurately stage diseases, localize lesions, and improve diagnostic efficiency during confocal endoscopic examinations.

This study has some limitations. The algorithm is not real-time because the stitching speed is slower than that of confocal endomicroscopy, resulting in a delay during actual examination. Second, as shown in Fig. 6, the pronounced gap in the Y-axis trajectory, the algorithm’s potential application is currently constrained by the lack of tracing technology specifically optimized for confocal endomicroscopy, where existing motion estimation frameworks exhibit incomplete compatibility with probe tracking characteristics, leading to difficulties in determining accurate stitched image contours and posing potential risks of contour deformation.

Future work will focus primarily on improving the real-time performance of the algorithm and integrating object tracking algorithms to confirm endoscope movement paths, generate accurate stitched image contours, and ultimately produce more informative stitched images. This will improve the efficiency and diagnostic accuracy for confocal endomicroscopy of the digestive tract.

## 5 Conclusion

In this paper, we propose a gland segmentation network-based stitching algorithm for the gastrointestinal CLE images. Our method leverages the unique textural features of gastrointestinal CLE images to generate precise gland segmentation masks, which are then employed to select stable frames and extract foreground features, thereby effectively suppressing rolling shutter effects and improving image stitching quality. Experimental results show that our proposed network outperforms several advanced deep learning segmentation methods in confocal endomicroscopic images of the gastrointestinal tract. Our image stitching method surpasses the AutoStitch method in accuracy and image quality. The resulting stitched images can display key diagnostic information, demonstrating significant potential for practical applications and clinical diagnosis support.

## Data Availability

The confocal endomicroscopy image dataset can be obtained from the authors upon reasonable request. The DGU-Net source code is available on the GitHub via the link https://github.com/PtK929/DGU-Net/tree/main.
